# Promoter methylation of *ITF2*, but not *APC*, is associated with microsatellite instability in two populations of colorectal cancer patients

**DOI:** 10.1186/s12885-016-2149-9

**Published:** 2016-02-17

**Authors:** Andrea J. Savio, Darshana Daftary, Elizabeth Dicks, Daniel D. Buchanan, Patrick S. Parfrey, Joanne P. Young, Daniel Weisenberger, Roger C. Green, Steven Gallinger, John R. McLaughlin, Julia A. Knight, Bharati Bapat

**Affiliations:** Department of Laboratory Medicine and Pathobiology, University of Toronto, Toronto, ON Canada; Lunenfeld-Tanenbaum Research Institute of Mount Sinai Hospital, Toronto, ON Canada; Ontario Familial Colorectal Cancer Registry, Toronto, ON Canada; Faculty of Medicine, Memorial University of Newfoundland, St John’s, Newfoundland, Canada; Oncogenomics Group, Genetic Epidemiology Laboratory, Department of Pathology, The University of Melbourne, Parkville, VIC Australia; Centre for Epidemiology and Biostatistics, Melbourne School of Population and Global Health, The University of Melbourne, Parkville, VIC Australia; Department of Haematology and Oncology, The Queen Elizabeth Hospital, Woodville, South Australia Australia; USC Epigenome Center, University of Southern California, Los Angeles, CA USA; Department of Surgery, University of Toronto, Toronto, ON Canada; Dalla Lana School of Public Health, University of Toronto, Toronto, ON Canada; Department of Pathology, University Health Network, Toronto, ON Canada

**Keywords:** Colorectal cancer, DNA methylation, Microsatellite instability, Wnt signaling, MethyLight

## Abstract

**Background:**

Aberrant Wnt signaling activation occurs commonly in colorectal carcinogenesis, leading to upregulation of many target genes. APC (adenomatous polyposis coli) is an important component of the β-catenin destruction complex, which regulates Wnt signaling, and is often mutated in colorectal cancer (CRC). In addition to mutational events, epigenetic changes arise frequently in CRC, specifically, promoter hypermethylation which silences tumor suppressor genes. *APC* and the Wnt signaling target gene *ITF2 (immunoglobulin transcription factor 2)* incur hypermethylation in various cancers, however, methylation-dependent regulation of these genes in CRC has not been studied in large, well-characterized patient cohorts. The microsatellite instability (MSI) subtype of CRC, featuring DNA mismatch repair deficiency and often promoter hypermethylation of *MutL homolog 1* (*MLH1*), has a favorable outcome and is characterized by different chemotherapeutic responses than microsatellite stable (MSS) tumors. Other epigenetic events distinguishing these subtypes have not yet been fully elucidated.

**Methods:**

Here, we quantify promoter methylation of *ITF2* and *APC* by MethyLight in two case-case studies nested in population-based CRC cohorts from the Ontario Familial Colorectal Cancer Registry (*n* = 330) and the Newfoundland Familial Colorectal Cancer Registry (*n* = 102) comparing MSI status groups.

**Results:**

*ITF2* and *APC* methylation are significantly associated with tumor versus normal state (both *P* < 1.0×10^-6^). *ITF2* is methylated in 45.8 % of MSI cases and 26.9 % of MSS cases and is significantly associated with MSI in Ontario (*P* = 0.002) and Newfoundland (*P* = 0.005) as well as the MSI-associated feature of *MLH1* promoter hypermethylation (*P* = 6.72×10^-4^). *APC* methylation, although tumor-specific, does not show a significant association with tumor subtype, age, gender, or stage, indicating it is a general tumor-specific CRC biomarker.

**Conclusions:**

This study demonstrates, for the first time, MSI-associated *ITF2* methylation, and further reveals the subtype-specific epigenetic events modulating Wnt signaling in CRC.

## Background

Colorectal cancer (CRC) is one of the most common cancers in the Western world and is marked by a high mortality rate [1]. Early detection of CRC is the key to improved survival rates [2]. Another factor affecting disease prognosis is CRC subtype [3]. The microsatellite instability (MSI) subtype of CRC accounts for approximately 15 % of colorectal cancers [4]. MSI tumors are distinguished by defects in the DNA mismatch repair system which leads to mutational insertions and deletions in short tandem repeats (microsatellites) of DNA [5]. MSI is most often due to promoter hypermethylation and silencing of the *MutL homolog 1* (*MLH1*) mismatch repair gene. Microsatellite stable (MSS) tumors account for 85 % of CRCs and exhibit chromosomal instability, including numerous chromosomal duplications, deletions and rearrangements [6]. MSI tumors differ from MSS tumors in several ways; MSI CRCs exhibit proximal colonic location, increased lymphocytic infiltration, and poorer response to chemotherapeutic drugs [7, 8]. MSI CRCs also demonstrate better prognosis at stages I-III, however, some studies suggest poor prognosis at stage IV, though metastatic MSI cases are rare [7, 9]. A third CRC subtype, the CpG island methylator phenotype (CIMP), is characterized by widespread DNA hypermethylation of CpG-rich promoter islands. CIMP can exist concurrently with either the MSI or MSS phenotype, though it is more frequently found in tandem with MSI and *MLH1* hypermethylation [10]. The prognostic significance of CIMP is currently undefined and may be modified by MSI status, presence of *BRAF* mutation, tumor stage, or other factors [11–13]. Recently, a classification system for further subtyping of CRC has been proposed, consisting of four subtypes [14]. One subtype consists mostly of MSI cases, while the other three are able to categorize the remainder of cases by Wnt signaling activation, metabolic dysregulation, or mesenchymal activation.

The vast majority (up to 94 %) of CRCs feature dysregulation in the Wnt signaling pathway [15]. Wnt signaling is important in normal development, cell growth and proliferation, but when inappropriately activated may also lead to tumor initiation and development [16]. In canonical Wnt signaling, β-catenin accumulates within the cell, enters the nucleus and activates transcription of target genes, such as c-Myc and *ITF2 (immunoglobulin transcription factor 2)* [17, 18]. *ITF2* is also known as *transcription factor 4* (*TCF4*). In the absence of Wnt signaling, a β-catenin destruction complex including adenomatous polyposis coli (APC) targets β-catenin for ubiquitination followed by proteasomal degradation [17, 18]. In many cases of CRC, the *APC* gene is mutated, rendering it incapable of binding to β-catenin, which leads to β-catenin accumulation followed by its nuclear translocation and subsequent activation of downstream target genes [18].

Evidence for DNA methylation of the *APC* promoter has been found in CRC. However, to what extent *APC* methylation plays a role in colorectal carcinogenesis is unclear, as a broad range of methylation levels has been found in the literature, from 11 up to 63 % of tumors methylated [19–23]. Conflicting reports exist regarding the extent of *APC* methylation in MSI CRCs. Some small-scale studies (MSI *n* ≤ 29) have suggested that *APC* methylation may be associated with the MSI subtype, but others show no significant difference [21–27]. Still another study has found *APC* methylation to be inversely correlated with CIMP but not MSI [28].

The role of *ITF2*, a Wnt signaling target gene, is less understood in CRC. It is a target of Wnt signaling and is overexpressed in colon cancers with Wnt dysregulation [29]. Its expression was reported elevated in some cancers with aberrant Wnt signaling activation but decreased in others [30, 31]. Among gastrointestinal malignancies, *ITF2* methylation has been reported in gastric cancer, but its methylation status has not been investigated in CRC [32, 33].

Our group has previously demonstrated associations between the methylation status of key Wnt signaling pathway regulatory genes and CRC subtype including the extracellular Wnt antagonists *DKK1* and *SFRP1* as well as *Wnt5a* which is involved in non-canonical Wnt activity [34, 35].

In this study, we have examined the role of *APC* and *ITF2* methylation in two nested case-case studies in CRC cohorts. These patients were recruited from two distinct Canadian populations and the case groups were stratified by their MSI status.

## Methods

### Study participants

Participants of this study were population-based primary CRC cases recruited through the Ontario Familial Colorectal Cancer Registry (OFCCR) and Newfoundland Familial Colorectal Cancer Registry (NFCCR). Procedures for patient accrual, biospecimen collection and data collection for the OFCCR and NFCCR have been previously described [36, 37]. Briefly, Ontario residents between the ages of 20 and 74 diagnosed with pathology-confirmed primary CRC between 1997 and 2000 were eligible for recruitment. Familial adenomatous polyposis cases were excluded and in the current study non-white patients were also excluded due to the high prevalence of self-reported Caucasians in the study (92.5 %). A total of 1168 participants have been analyzed for MSI status (see Molecular analysis below) of which 184 are MSI high (MSI-H). 165 of these MSI-H cases had available DNA of high quality. A matched case-case selection strategy was utilized to select 165 patients with MSS tumors to match 165 patients with MSI-H tumors by sex, stage at diagnosis and age quartile. The 165 MSS tumors were selected from a total of 384 MSS tumors available at the time this study was undertaken. Population-based recruitment by the NFCCR was similar to the OFCCR, with a recruitment period from 1999 to 2003 of cases from provincial tumor registries [37]. For the NFCCR, proxy consent from living family members was obtained for deceased patients leading to a high frequency of late-stage patients. These tumor samples were not utilized in order to maintain similar patient age and tumor stage between the Ontario and Newfoundland populations. 102 tumor samples from 696 total CRC cases were chosen from probands of the NFCCR, 51 of which were MSI-H, matched to 51 MSS cases by sex, stage at diagnosis and age quartile. Overall survival status, along with other patient clinicopathological features, is described in Table 1. Recurrence data was not available for all cases used in this study, thus was not included in analysis. DNA from normal colonic mucosa was also available for all patients. Of the 330 OFCCR and 102 NFCCR patients’ tumor samples utilized, 47 were selected randomly for methylation analysis of normal adjacent tissue. Patient data was obtained through protocols approved by the Research Ethics Boards of Mount Sinai Hospital, the University of Toronto and Memorial University of Newfoundland. All patients or their proxies provided informed consent.

**Table 1:** Clinicopathological features of primary colorectal carcinomas of patients from Ontario and Newfoundland

	No. of cases (%)			
	Ontario		Newfoundland	
	MSS	MSI-H	MSS	MSI-H
Cases of primary colorectal carcinoma	165	165	51	51
Mean age (±SD^a^)	59.9 (9.3)	60.1 (9.8)	58.3 (10.2)	58.4 (10.2)
Age				
<50	19 (11.5)	28 (17.0)	11 (21.6)	10 (19.6)
50+	146 (88.5)	137 (83.0)	40 (78.4)	41 (80.4)
Sex				
Male	74 (44.8)	74 (44.8)	26 (51.0)	26 (51.0)
Female	91 (55.2)	91 (55.2)	25 (49.0)	25 (49.0)
TNM Stage				
1	38 (23.0)	38 (23.0)	12 (23.5)	11 (21.6)
2	84 (50.9)	85 (51.5)	26 (51.0)	27 (52.9)
3	34 (20.6)	39 (23.6)	10 (19.6)	12 (23.5)
4	9 (5.5)	3 (1.8)	3 (5.9)	1 (2.0)
Histological Grade				
Low	16 (9.7)	10 (6.1)	4 (7.8)	7 (13.7)
Moderate	123 (74.5)	45 (27.3)	37 (72.6)	36 (70.6)
High	13 (7.9)	20 (12.1)	8 (15.7)	8 (15.7)
Unavailable	13 (7.9)	90 (54.5)	2 (3.9)	
Location^b^				
Distal	108 (65.5)	15 (9.1)	37 (72.5)	9 (17.6)
Proximal	51 (30.9)	63 (38.2)	14 (27.5)	42 (82.4)
Unavailable	6 (3.6)	87 (52.7)		
Histological Type^c^				
Non-Mucinous	143 (86.7)	107 (64.8)	46 (90.2)	42 (82.4)
Mucinous	19 (11.5)	53 (32.1)	5 (9.8)	9 (17.6)
Unavailable	3 (1.8)	5 (3.0)		
MMR Protein Status				
Intact	148 (89.7)	26 (15.8)	50 (98.0)	4 (7.8)
Deficient	4 (2.4)	136 (82.4)	0 (0.0)	46 (90.2)
Unavailable	13 (7.9)	3 (1.8)	1 (2.0)	1 (2.0)
MMR Germline Mutation				
No	164 (99.4)	124 (75.2)	51 (100.0)	39 (76.5)
Yes	1 (0.6)	41 (24.8)	0 (0.0)	12 (23.5)
*MLH1* Methylation				
Unmethylated	159 (96.4)	87 (52.7)	36 (70.6)	23 (45.1)
Methylated	5 (3.0)	78 (47.3)	1 (2.0)	28 (54.9)
Unavailable	1 (0.6)		14 (27.4)	
BRAF V600E Mutation				
No	146 (88.5)	95 (57.6)	46 (90.2)	25 (49.0)
Yes	15 (9.1)	66 (40.0)	2 (3.9)	20 (39.2)
Unavailable	4 (2.4)	4 (2.4)	3 (5.9)	6 (11.8)
CIMP Status				
Negative	133 (80.6)	79 (47.9)		
Positive	10 (6.1)	63 (38.2)		
Unavailable	22 (13.3)	23 (13.9)	51 (100.0)	51 (100.0)
Survival Status				
Alive	100 (60.6)‬	99 (60.0)	45 (88.2)	49 (96.1)
Deceased	65 (39.4)	66 (40.0)	6 (11.8)	2 (3.9)
*ITF2* Methylation				
Unmethylated	122 (73.9)	95 (57.6)	36 (70.6)	22 (43.1)
Methylated	43 (26.1)	70 (42.4)	15 (29.4)	29 (56.9)
*APC* Methylation				
Unmethylated	112 (67.9)	103 (67.9)	33 (64.7)	28 (54.9)
Methylated	53 (32.1)	62 (37.6)	18 (35.3)	23 (45.1)

^a^Standard Deviation

^b^Proximal tumor location includes lesions up to and including the splenic flexure

^c^Mucinous histology includes the presence of any mucin within the tumor stroma

### Molecular analysis

DNA used to assess MSI status was extracted from archival paraffin-embedded tumors microdissected to enrich for tumor cells. MSI status was assessed using National Cancer Institute guidelines using four or more of the following markers: ACTC, BAT-25, BAT-26, BAT-40, BAT-34C4, D10S197, D18S55, D17S250, D5S346 and MYC-L. The numbers of positive markers used to define MSI status are: MSI high (MSI-H), ≥30 % unstable markers; MSI low (MSI-L), 1–29 % unstable markers; MSS, 0 % unstable markers [38]. Tumors with MSI-L status were not included in this study.

Somatic T > A mutation of nucleotide 1799 in the *BRAF* gene leading to the V600E mutation was determined by allele-specific PCR as described previously [34].

Immunohistochemistry was used to determine presence of the mismatch repair proteins MLH1, MSH2, MSH6 and PMS2. Protein staining was classified as either present, absent, or inconclusive. Tumors without positive staining for any of these proteins were defined as mismatch repair deficient, as described previously [39].

### MethyLight analysis

The sensitive, semi-quantitative high-throughput MethyLight assay was used to analyze the methylation of *APC* and *ITF2* in tumor and normal colonic DNA of CRC patients. DNA was treated with sodium bisulfite prior to MethyLight according to protocol using the EZ DNA Methylation Gold Kit (Zymo Research Corp, Orange, CA). Primers and probe were used to amplify a region within the CpG island of promoter 1A of *APC*. Forward primer: 5′-GAACCAAAACGCTCCCCAT-3′. Probe: 5′-CCCGTCGAAAACCCGCCGATTA-3′. Reverse primer: 5′-TTATATGTCGGTTACGTGCGTTTATAT-3′. Primers and probe were designed within the promoter region of *ITF2.* Forward primer: 5′-GAAGCGGTAATACGAATAAGAGC-3′. Probe: 5′-ATTCCCGAAACCGAAATCGTTCGCAAACC-3′. Reverse primer: 5′- AACTATTCTCGAATAAACGTCGC-3′. Alu-C4 was also amplified to normalize the DNA input. Forward primer: 5′-GGTTAGGTATAGTGGTTTATATTTGTAATT-3′. Probe: 5′-CCTACCTTAACCTCCC-3′. Reverse primer: 5′-ATTAACTAAACTAATCTTAAACTCCTA-3′. Probes contained a 5′ fluorescent reporter dye and a 3′ quencher dye. Samples were analyzed using the ABI 7500 RT-PCR thermocycler in 96-well plates as previously described [40]. *APC*, *ITF2* and Alu-C4 were also amplified in exogenously methylated CpGenome DNA (Millipore, Billerica, MA). The percent methylated reference (PMR) was calculated to assess the methylation using the formula: (Gene of Interest/Alu-C4)_sample_/(Gene of Interest/Alu-C4)_CpGenome_x100%. In order to ensure that DNA quality was adequate, samples with an Alu-C4 threshold cycle greater than 22 were deemed poor quality and re-analyzed or removed from the study [41].

*MLH1* methylation status was assessed by MethyLight as described previously, with positive methylation defined as PMR ≥ 10 % [42]. CIMP status was determined using the Weisenberger panel of markers, described previously [43]. Briefly, MethyLight was used to assess a 5-gene signature consisting of *CACNA1G, IGF2, NEUROG1, RUNX3* and *SOCS1*. Tumors were classified as CIMP if 3 or more of 5 genes had PMR ≥ 10 % and non-CIMP if 2 or fewer genes had PMR ≥ 10 %. CIMP status was available for a subset of Ontario cases (285 of 330) and unavailable for Newfoundland cases.

### Statistical analysis

Comparison of the methylation status of matched tumor and normal DNA samples was performed using McNemar’s test. Results were considered statistically significant if two-sided *P* < 0.05. Pearson’s chi-square test was used to measure associations between clinicopathological variables and *ITF2* and *APC* methylation in tumor DNA. Bonferroni correction was used to account for multiple comparisons. All analyses were performed using PASW Statistics 21 (SPSS Inc., Chicago, IL).

## Results

### ITF2 promoter methylation in CRC tumors and normal colonic mucosa

Patient clinicopathological features are shown in Table 1 for MSI-H and MSS cases for the Ontario and Newfoundland populations. We quantified promoter methylation of *ITF2* using MethyLight in CRC tumors from Ontario and Newfoundland. To quantify *ITF2* promoter methylation levels in both normal mucosa and CRC tumor tissue we tested 47 randomly selected normal-tumor pairs from Ontario and Newfoundland CRC cases, of which 12 were MSI-H and 35 were MSS. The mean PMR was 8.8 % in tumor DNA and 1.6 % in normal colonic DNA. Methylation levels ranged from 0–31.1 % in tumor and 0–15.6 % in normal colonic DNA. A PMR cut-off of 10 % was used to dichotomize methylated and unmethylated samples. McNemar’s test comparing methylation above this cut-off in tumor and normal tissues in CRC patients determined tumor methylation of *ITF2* to be significantly higher than normal colonic mucosa methylation (*P* < 1.0×10^-6^). For *ITF2* promoter methylation, comparable methylation levels were seen in CRC tumors between the two populations, comprised of 165 MSI-H and 165 MSS cases from Ontario and 51 MSI-H and 51 MSS cases from Newfoundland. In the Ontario cases the mean PMR for all 330 cases was 8.5 %. Methylation values ranged from 0–57.2 %. Using the PMR cut-off of 10 %, 34.2 % (113/330) of cases were considered positively methylated in the Ontario cohort. In Newfoundland cases the mean PMR for all 102 cases was 15.4 %. Methylation values ranged from 0–95.4 %. Using the PMR cut-off of 10 %, 43.1 % (44/102) of cases were considered positively methylated in the Newfoundland cohort.

### APC promoter methylation in CRC tumors and normal colonic mucosa

We quantified methylation of the *APC* promoter region in tumor and matched normal colonic mucosa of 47 randomly selected patients from both Ontario and Newfoundland. Mean methylation was 16.1 % in tumor DNA and 2.6 % in normal colon. Methylation values ranged from 0–60.2 % for tumor samples and from 0–11.9 % for normal samples. A PMR cut-off of 10 % was used to dichotomize methylated and unmethylated samples. McNemar’s test comparing methylation above this cut-off in tumor and normal tissues in CRC patients determined tumor methylation of *APC* to be significantly higher than normal colonic mucosa methylation (*P* < 1.0×10^-6^).

For APC promoter methylation, comparable methylation levels were seen in CRC tumors between the two populations. The mean PMR in Ontario was 11.5 %. Methylation values ranged from 0–92.2 %. For the Newfoundland samples, the mean PMR was 13.9 %. Methylation values ranged from 0–70.5 %. Using the PMR cut-off of 10 %, 34.8 % of tumors (115/330) were methylated in the Ontario cohort and 40.2 % of tumors (41/102) were methylated in the Newfoundland cohort.

### ITF2 methylation and clinicopathological features, including MSI subtype, in two distinct CRC cohorts

We examined whether methylation of *ITF2* in tumor DNA was associated with patient clinicopathological features. Methylation status was compared by Pearson’s chi-square test between MSI-H and MSS cases. In Ontario 26.1 % (43/165) of MSS cases compared to 42.4 % (70/165) of MSI-H cases were methylated, with an odds ratio (OR) of 2.09 [95 % confidence interval (CI) 1.31–3.33], and *P* = 0.002. Similarly in Newfoundland 29.4 % (15/51) of MSS cases and 56.9 % (29/51) of MSI-H cases were methylated with OR of 3.16 (95 % CI 1.40–7.17), and *P* = 0.005. Ontario and Newfoundland data was then pooled for further analysis.

Due to our selection strategy and the association between MSI and *ITF2* methylation, further clinicopathological associations were analyzed separately for MSI-H and MSS cases, shown in Table 2. There was a significant association between *ITF2* promoter methylation with *MLH1* promoter methylation in MSI-H cases, *P* = 6.72×10^-4^, OR 1.88 (1.10–3.68). There was also a trend towards an association between *ITF2* methylation and female gender in MSI-H cases, and *ITF2* methylation and CIMP in MSS cases, but this was not significant using a conservative *p*-value adjusted for multiple comparisons. No other significant associations were found for either MSS or MSI-H cases between *ITF2* methylation and early age of onset (<50 years), stage, grade, tumor location, histological type, MMR protein status, MMR germline mutation, BRAF V600E mutation, or survival status. Stage-specific survival was also performed, to account for potential differences in early stage survival compared to stage IV in MSI-H cases (data not shown). There was a trend toward higher overall survival in stage I MSI-H cases with *ITF2* methylation, but results were not significant after correction for multiple comparisons.

**Table 2:** Associations between *ITF2* methylation and clinicopathological features in tumor DNA. Analysis of 216 MSI-H and 216 MSS CRC patients from Ontario and Newfoundland

	MSS (*n* = 216)				MSI-H (*n* = 216)			
	Unmethylated (%)	Methylated (%)	OR (95 % CI)^a^	*P*-value	Unmethylated (%)	Methylated (%)	OR (95 % CI)^a^	*P*-value
Age								
<50	24 (15.2)	6 (10.3)	1.552 (0.600–4.014)	0.361	24 (20.5)	14 (14.1)	1.567 (0.761–3.225)	0.220
50+	134 (84.8)	52 (89.7)			93 (79.5)	85 (85.9)		
Sex								
Male	73 (46.2)	26 (44.8)	1.057 (0.577–1.935)	0.857	64 (54.7)	36 (36.4)	2.113 (1.222–3.655)	0.007
Female	85 (53.8)	32 (55.2)			53 (45.3)	63 (63.6)		
TNM Stage^b^								
1	36 (22.8)	14 (24.1)	0.720 (0.606–0.856)	0.193	31 (26.5)	18 (18.2)	5.167 (0.499–53.450)	0.321
2	78 (49.4)	32 (55.2)			57 (48.7)	55 (55.6)		
3	32 (20.3)	12 (20.7)			28 (23.9)	23 (23.2)		
4	12 (7.6)	0 (0.0)			1 (0.9)	3 (3.0)		
Histological Grade^b^								
Low	16 (10.8)	4 (7.5)	2.000 (0.482–8.295)	0.624	9 (13.8)	8 (13.1)	0.728 (0.215–2.459)	0.514
Moderate	118 (79.7)	42 (79.2)			39 (60.0)	42 (68.9)		
High	14 (9.5)	7 (13.2)			17 (26.2)	11 (18.0)		
Location^c^								
Proximal	58 (37.9)	29 (50.9)	0.589 (0.319–1.089)	0.090	60 (89.6)	54 (87.1)	1.270 (0.432–3.735)	0.664
Distal	95 (62.1)	28 (49.1)			7 (10.4)	8 (12.9)		
Histological Type^d^								
Non-Mucinous	140 (90.3)	49 (84.5)	1.714 (0.705–4.167)	0.230	76 (66.7)	73 (75.3)	0.658 (0.360–1.202)	0.172
Mucinous	15 (9.7)	9 (15.5)			38 (33.3)	24 (24.7)		
MMR Protein Status								
Intact	143 (97.9)	55 (98.2)	0.867 (0.088–8.511)	0.902	21 (18.6)	9 (9.1)	2.283 (0.992–5.251)	0.048
Deficient	3 (2.1)	1 (1.8)			92 (81.4)	90 (90.9)		
MMR Germline Mutation								
No	157 (99.4)	58 (100.0)	0.730 (0.673–0.792)	0.544	89 (76.1)	74 (74.7)	1.074 (0.577–1.999)	0.822
Yes	1 (0.6)	0 (0.0)			28 (23.9)	25 (25.3)		
*MLH1* Methylation								
Unmethylated	143 (96.0)	52 (100.0)	0.733 (0.674–0.798)	0.142	68 (58.1)	42 (42.4)	1.883 (1.095–3.238)	6.72x10^-4^
Methylated	6 (4.0)	0 (0.0)			49 (41.9)	57 (57.6)		
BRAF V600E Mutation								
No	141 (92.2)	51 (91.1)	1.152 (0.387–3.431)	0.799	70 (63.1)	50 (52.6)	1.537 (0.088–2.683)	0.130
Yes	12 (7.8)	5 (8.9)			41 (36.9)	45 (47.4)		
CIMP Status								
Negative	100 (96.2)	33 (84.6)	4.545 (1.208–17.100)	0.016	50 (61.7)	29 (47.5)	1.780 (0.908–3.489)	0.092
Positive	4 (3.8)	6 (15.4)			31 (38.3)	32 (52.5)		
Survival Status								
Alive	110 (69.6)	35 (60.3)	1.475 (0.790–2.755)	0.221	78 (66.7)	70 (70.7)	0.829 (0.464–1.478)	0.524
Deceased	48 (30.4)	23 (39.7)			39 (33.3)	29 (29.3)		

^a^Odds ratio and 95 % confidence interval for methylated versus unmethylated

^b^OR and 95 % CI given for lowest stage/grade versus highest stage/grade

^c^Proximal tumor location includes lesions up to and including the splenic flexure

^d^Mucinous histology includes the presence of any mucin within the tumor stroma

### APC methylation and clinicopathological features, including MSI subtype, in two distinct CRC cohorts

We examined whether methylation of *APC* in tumor DNA was associated with patient clinicopathological features in both cohorts. Methylation status was compared by Pearson’s chi-square test between MSI-H and MSS cases. In Ontario 32.1 % (53/165) of MSS cases were methylated while 37.6 % (62/165) of MSI-H cases were methylated with an OR (95 % CI) of 1.27 (0.81–2.00), and *P* = 0.298. Similarly in Newfoundland 35.3 % (18/51) of MSS cases were methylated and 45.1 % (23/51) of MSI-H cases methylated with OR (95 % CI) of 1.51 (0.68–3.34), and *P* = 0.313.

We examined whether *APC* methylation was associated with patient clinicopathological features in pooled CRC cases from Ontario and Newfoundland. Results are shown in Table 3. Methylation of *APC* was not found to be associated with early age of onset (<50 years), sex, stage, grade, tumor location, histological type, CIMP status, MMR protein status, MMR germline mutation, *MLH1* methylation, BRAF V600E mutation, or survival status.

**Table 3:** Associations between *APC* methylation and clinicopathological features in tumor DNA. Analysis of 216 MSI-H and 216 MSS CRC patients from Ontario and Newfoundland

	MSS (*n* = 216)				MSI-H (*n* = 216)			
	Unmethylated (%)	Methylated (%)	OR (95 % CI)^a^	*P*-value	Unmethylated (%)	Methylated (%)	OR (95 % CI)^a^	*P*-value
Age								
<50	20 (13.8)	10 (14.1)	0.976 (0.431–2.213)	0.954	23 (17.6)	15 (17.9)	0.994 (0.485–2.035)	0.986
50+	125 (86.2)	61 (85.9)			108 (82.4)	70 (82.4)		
Sex								
Male	64 (44.1)	35 (49.3)	0.813 (0.460–1.436)	0.475	59 (45.0)	41 (48.2)	0.879 (0.509–1.520)	0.645
Female	81 (55.9)	36 (50.7)			72 (55.0)	44 (51.8)		
TNM Stage^b^								
1	27 (18.6)	23 (32.4)	0.391 (0.095–1.619)	0.147	32 (24.4)	17 (20.0)	0.653 (0.533–0.801)	0.306
2	79 (54.5)	31 (43.7)			64 (48.9)	48 (56.5)		
3	30 (20.7)	14 (19.7)			31 (23.7)	20 (23.5)		
4	9 (6.2)	3 (4.2)			4 (3.1)	0 (0.0)		
Histological Grade^b^								
Low	12 (8.9)	8 (12.1)	0.600 (0.163–2.207)	0.724	8 (11.0)	9 (17.0)	0.770 (0.230–2.578)	0.467
Moderate	108 (80.0)	52 (78.8)			50 (68.5)	31 (58.5)		
High	15 (11.1)	6 (9.1)			15 (20.5)	13 (24.5)		
Location^c^								
Proximal	62 (44.0)	25 (36.2)	1.381 (0.763–2.499)	0.285	63 (85.1)	51 (92.7)	0.449 (0.135–1.495)	0.183
Distal	79 (56.0)	44 (63.8)			11 (14.9)	4 (7.3)		
Histological Type^d^								
Non-Mucinous	129 (89.6)	60 (87.0)	1.290 (0.534–3.114)	0.570	87 (68.5)	62 (73.8)	0.772 (0.418–1.426)	0.408
Mucinous	15 (10.4)	9 (13.0)			40 (31.5)	22 (26.2)		
MMR Protein Status								
Intact	132 (97.8)	66 (98.5)	0.668 (0.068–6.533)	0.726	21 (16.3)	9 (10.8)	1.599 (0.694–3.685)	0.268
Deficient	3 (2.2)	1 (1.5)			108 (83.7)	74 (89.2)		
MMR Germline Mutation								
No	144 (99.3)	71 (100.0)	0.670 (0.610–0.736)	0.483	104 (79.4)	59 (69.4)	1.697 (0.908–3.175)	0.096
Yes	1 (0.7)	0 (0.0)			27 (20.6)	26 (30.6)		
*MLH1* Methylation								
Unmethylated	131 (96.3)	64 (98.5)	0.409 (0.047–3.577)	0.405	62 (47.3)	48 (56.5)	0.693 (0.400–1.199)	0.189
Methylated	5 (3.7)	1 (1.5)			69 (52.7)	37 (43.5)		
BRAF V600E Mutation								
No	126 (90.6)	66 (94.3)	0.587 (0.184–1.873)	0.364	67 (53.2)	53 (66.3)	0.579 (0.324–1.034)	0.064
Yes	13 (9.4)	4 (5.7)			59 (46.8)	27 (33.8)		
CIMP Status								
Negative	91 (92.9)	42 (93.3)	0.929 (0.229–3.770)	0.917	45 (52.3)	34 (60.7)	0.710 (0.359–1.406)	0.325
Positive	7 (7.1)	3 (6.7)			41 (47.7)	22 (39.3)		
Survival Status								
Alive	95 (65.5)	50 (70.4)	0.782 (0.424–1.444)	0.432	91 (69.5)	57 (67.1)	1.118 (0.622–2.007)	0.710
Deceased	50 (34.5)	21 (29.6)			40 (30.5)	28 (32.9)		

^a^Odds ratio and 95 % confidence interval for methylated versus unmethylated

^b^OR and 95 % CI given for lowest stage/grade versus highest stage/grade

^c^Proximal tumor location includes lesions up to and including the splenic flexure

^d^Mucinous histology includes the presence of any mucin within the tumor stroma

## Discussion

Understanding the genetic and epigenetic differences amongst colorectal cancer subtypes is essential, as CRC subtypes differ in their treatment options and offer distinct survival outcomes. The Wnt signaling pathway is dysregulated in a majority of colorectal tumors and can be altered at the extracellular, intracellular and gene target level [15, 34, 35]. We have shown that these changes to the Wnt pathway also differ based upon microsatellite instability status. We quantified the methylation status of the *ITF2* and *APC* promoter CpG islands in a nested case-case study in two cohorts of colorectal carcinoma from two different populations, comparing cases by MSI status. We have demonstrated that the *ITF2* promoter is hypermethylated in tumor tissues compared with matched normal mucosa, and further, MSI-H tumors are more likely to incur promoter methylation compared with MSS tumors. *ITF2* promoter methylation was also significantly associated with *MLH1* promoter methylation, a common occurrence in MSI-H tumors. Conversely, we found that *APC,* an important intracellular regulator of Wnt signaling marked by both genetic mutations and hypermethylation in CRC, acquires DNA methylation equally across subtypes.

This is the first study to investigate DNA methylation of *ITF2* in CRC cases. Here, we have established that *ITF2* methylation is a tumor-associated event, being a rare occurrence in normal tissue DNA. One sample out of 47 normal colonic tissue samples was methylated, but this rare occurrence may possibly be due to the field effect, or field cancerization, in which apparently normal cells acquire genetic and/or epigenetic alterations and may eventually progress to cancer. With regards to tumor methylation of *ITF2*, we showed that it is associated with the MSI-H phenotype. *ITF2* has been reported to be a tumor suppressor that can induce cell cycle arrest and is sometimes lost due to loss of heterozygosity at 18q21 [31]. However, *ITF2* expression has been found to be upregulated in some cancers with aberrantly activated Wnt signaling but decreased in others [30, 31]. Further research is required to elucidate the role of *ITF2* in tumorigenesis. Treatment of gastric cancer cell lines with the DNA methyltransferase inhibitor 5-aza-2’-deoxycytidine (5-aza) restored mRNA expression in cell lines that had hypermethylation demonstrating methylation-dependent regulation of this gene [33]. Thus, transcriptional silencing in CRC through methylation would likely lead to a decrease in its cellular expression levels potentially contributing to tumorigenesis.

*APC* promoter methylation is rarely observed in normal colonic tissue compared with CRC tumor tissue in our study population, which replicates the findings of a recent meta-analysis [44]. However, contrary to *ITF2* methylation, we did not see an association with MSI-H CRC or any other clinical features. *APC* expression is at least partially regulated by DNA methylation, as its expression increases in CRC cell lines after treatment with 5-aza [45]. Several other studies have investigated the correlation between *APC* methylation and MSI with varying results. Studies have shown wide variation in overall APC methylation, regardless of subtype, from as low as 18 % to as high as 63.4 % [19, 20]. Our results show a more moderate level of 34–40 % of cases methylated. Findings in the literature for the correlation between *APC* and MSI are even less clear, with methylation in MSI-H tumors ranging from 14.3–72.7 % [21, 26]. However, these studies analyzed small numbers of patient samples, with a maximum of 29 MSI tumors used [24]. Our study, on the other hand, employed a total of 432 samples, 216 of which were MSI-H. This sample size is many times larger than any other of its kind, giving more statistical power and certainty to our results.

There are no differences between level of methylation at different stages of CRC diagnosis for either *APC* or *ITF2*, indicating these may be early epigenetic events in tumorigenesis. Additionally, *APC* methylation has been detected in colon adenoma, further evidence that it is an early event [46]. Detection of *APC* may be further exploited as a potential biomarker by detection in other biospecimens, as its methylation has been detected in both stool and plasma [47, 48]. Further investigation of the presence of *ITF2* methylation in adenomas should be undertaken, as well as whether its methylation can be detected in stool or plasma. This research will indicate the potential of utilizing *ITF2* and *APC,* perhaps in combination with other methylation markers, as non-invasive stool- or plasma-based methylation markers for CRC detection and/or subtype discrimination.

Data from colon and rectal tumors from The Cancer Genome Atlas (TCGA) shows that *APC* mutation rates differ among the 224 tumors sequenced by exome sequencing. TCGA data described hypermutated tumors, which have a mutation rate of 12/10^6^ and consist mostly of MSI-H tumors. The prevalence of *APC* mutation in these hypermutated tumors is 51 % [15]. Alternatively, non-hypermutated tumors, defined by a mutation rate <8.24/10^6^ and consisting mostly of MSS tumors, incurred *APC* mutations in 81 % of cases [15]. This disparity in *APC* mutation rates may be explained by DNA methylation to inactivate *APC* leading to constitutive ligand-independent Wnt signaling. In this same data set *ITF2* is genetically altered in only 3 % of tumors, thus, methylation is likely to play a larger role in *ITF2* dysregulation in cancer [49, 50].

While MSI-H tumors are a largely well-defined subtype of CRC, MSS tumors comprise the majority of cases and exhibit a wide variety of molecular characteristics. Thus, there is an emerging research focus to further classify molecular subtypes of CRC. Recently, four consensus molecular subtypes were defined. The first subtype consists mostly of MSI cases [14]. The remaining three subtypes are defined by ‘canonical’ Wnt and MYC activation, metabolic dysregulation, or mesenchymal activation. Our results indicated that some MSS cases incur methylation of the Wnt genes studied, so perhaps these cases belong to the subtype characterized by Wnt activation. It would be interesting to see which sub-classification the MSS cases used in this study belong to, and how *ITF2* or *APC* methylation profiles differ among the four subtypes.

MSI-H tumors often overlap with CIMP-positive status. Thus, the association we see between MSI-H and *ITF2* methylation may in fact be part of the widespread hypermethylation of CpG islands that characterizes CIMP tumors. CIMP status information is unavailable for some Ontario cases and all Newfoundland cases utilized in this study, thus we do not have a complete picture of CIMP for our cohort. From our available data we did see a trend between CIMP-positive status and *ITF2* methylation among MSS cases. However, there were only ten cases in this group. From our current findings as well as previous investigation into epigenetic regulation of Wnt signalling genes we have found that dysregulation through aberrant methylation is implicated in all subtypes of CRC, not solely in CIMP-positive cases. APC, which abrogates Wnt signaling intracellularly, is methylated in a proportion of CRCs, regardless of subtype while *ITF2,* a downstream target of Wnt signaling, is methylated more often in MSI-H tumors. Our lab has previously found that *DKK1* and *SFRP1* promoter methylation, coding for two extracellular Wnt antagonists, segregate strongly with different CRC subtypes. *DKK1* methylation is associated with the MSI-H phenotype and other MSI-associated features, while *SFRP1* methylation is associated with MSS tumors [34]. We also found that *Wnt5a* methylation, which codes for an extracellular ligand of the non-canonical Wnt pathway, is associated with MSI-H [35]. These results were found in the same cohort of Ontario and Newfoundland patients used in this study. These observations underscore the importance of both Wnt signaling and the role of DNA methylation in CRC.

One limitation of this study to bear in mind is that only a subset of available MSS cases was chosen for analysis by matching to MSI-H cases by age quartile, stage and sex. Individuals with MSI-H CRC are generally a younger age, more frequently female, have a lower tumor stage and are more frequently CIMP-positive than those individuals with MSS tumors. Thus, the MSS cases analyzed in this study do not wholly represent all MSS cases from our Ontario and Newfoundland populations. Additionally, we did not select MSS cases from the entire Ontario cohort, but only a subset available at the time this study was undertaken. The subset that we selected from did not differ in age, sex, stage or CIMP rates from the entire cohort.

The strengths of our study include large sample size, the inclusion of two independent well-characterized population-based cohorts and the choice of technology. The use of MethyLight technology is superior to methylation-specific PCR (MSP) and offers several advantages including a quantitative, high-throughput methylation-specific real-time PCR-based technique, which is amenable to using small quantities of DNA extracted from archival tissue specimens. MSP is a more qualitative and subjective method that has been used in many prior studies of *APC* methylation.

## Conclusions

Our findings demonstrate the importance of DNA methylation in the regulation of genes selected for analysis and its differing effects based on tumor subtype. *ITF2* is not yet well studied in CRC and we have now shown that this gene incurs MSI-associated hypermethylation. For *APC,* both mutation and methylation play a role in its dysregulation. It is likely that methylation of *APC* plays a secondary role in CRC to more commonly occurring mutations and may act to fine-tune Wnt signaling. With both mutation and methylation contributing to regulation of this gene, it is possible the sequence of events may dictate the way CRC evolves. Based on its high specificity for CRC, *APC* methylation may offer usefulness as a marker within a panel of other genes for CRC detection and *ITF2* may be useful for detection of MSI-H tumors. Future studies to independently validate these findings are warranted. Overall, this study has investigated methylation of the Wnt genes *APC* and *ITF2* in a large cohort of MSI-H and matched MSS CRC tumors to find that *ITF2* methylation is significantly associated with MSI-H tumors while *APC* methylation is a tumor-specific event in CRC, which does not differ significantly between MSI-H and MSS subtypes or other clinicopathological variables.

## Abbreviations

5-aza: 5-aza-2’-deoxycytidine
APC: adenomatous polyposis coli
CI: confidence interval
CIMP: CpG island methylator phenotype
CRC: colorectal cancer
DKK1: dickkopf 1 homolog [Xenopus laevis]
ITF2: immunoglobulin transcription factor 2/transcription factor 4
MLH1: MutL homolog 1
MSI: microsatellite instability
MSI-H: microsatellite instability high
MSI-L: microsatellite instability low
MSP: methylation-specific polymerase chain reaction
MSS: microsatellite stable
NFCCR: Newfoundland Familial Colorectal Cancer Registry
OFCCR: Ontario Familial Colorectal Cancer Registry
OR: odds ratio
PMR: percent methylated reference
SD: standard deviation
SFRP1: secreted frizzled-related protein 1
TCGA: The Cancer Genome Atlas
Wnt5a: wingless-type MMTV integration site family, member 5A
